# Proceedings

**Published:** 1889-12

**Authors:** 


### PROCEEDINGS.

### Fifth Annual Meeting of the Ohio State Dental Society, Hollenden House, Cleveland, Ohio, Oct., 29, 1889.

Society called to order at 10:30 A. M. by Pres. C. M. Wright. Prayer was offered by Rev. Wm. Sampson, Supt. of Children's Home, Cleveland, O.

Minutes of previous session read and approved. Dr. J. E. Robinson made a verbal report on behalf of Committee of Arrangements. Report accepted.

On motion of Prof. J. Taft a Committee on Necrology was appointed by the president. The following committee was appointed: Drs. J. Taft, H. A. Smith, C. R. Butler.

On motion of Prof. H. A. Smith a courteous greeting was extended to the representatives of the various district societies and they were tendered the privilege of the floor.

Moved by Prof. Smith that the society now hear reports from the district societies. On motion of Dr. Butler, the above motion was laid on the table.

On motion of Dr. Sillito, dental dealers were requested to close their rooms during meeetings of the society. Adjourned to 2 P. M.

ARTERNOON SESSION.

October 29, 1889. Society called to order by president at 2:30 P. M. Minutes of last session read and approved.

The following named applicants, having been duly recommended by the Board of Directors, were severally elected to active membership in this society : Dr. W. D. Kempton, Cincinnati ; Dr. Martha J. Robinson, Cleveland ; Dr. J. W. Jungman, Cleveland.

The society then listened to the President's address, in which he advocated the necessity of revising the present dental law. After a lengthy discussion, Dr. J. Taft moved that a committee of three be appointed to consider the status of the present law and report to this society.

President appointed the following committee: Drs. J. Taft,

J. E. Robinson, Otto Arnold. Moved by Dr. Taft that the report of the above committee be heard to-morrow at 11 o'clock. Motion carried.

Dr. H. H. Harrison's paper, Subject, Excelsior, was then read by Dr. J. E. Robinson; paper discussed by Dr. Smith and others.

A paper was then read by Dr. C. R. Butler. Subject, Failures. Paper discussed at length by Drs. Kempton, Smith, Robinson and others. On motion, the subject was passed.

Moved to adjourn to 9 o'clock to-morrow morning. Carried.

MORNING SESSION.

October 30, 1889. Society called to order by President at 10 A. M. Minutes of last session read and approved.

A petition was presented to place Dr. H. H. Harrison on the list of Honorary Membership. After discussion it was decided to lay the petition on the table, for the reason that Dr. Harrison is still a resident of the State and has an office at Cadiz, Ohio.

The following named applicants, having been duly recommended by the Board of Directors, were severally elected to active membership: Dr. F. H. Lyder, Dr. F. E. Battershall, Dr. G. H. Owens, Dr. P. S. Bollinger.

Committee on dental law offered the following report:

Your committee to whom was referred the matter of securing proper legislation for the regulation of the practice of dentistry in the State of Ohio, would report that after looking carefully over the ground, will suggest, that the wise and more practicable plan is to ask the Legislature to amend the present law, rather than to apply for a new enactment, and that these amendments consist of the addition of a clause or section requiring and providing for the registration of all legal practitioners who may now or hereafter be in practice in the State. And furthermore, that the penalty clause, which was omitted in the codification of the law, be restored. Your committee would further suggest that a committee of three be appointed to have this matter in charge and see that it is properly brought before the Legislature, and to employ all proper means, according to their judgment, that may be necessary for the accomplishment of the desired object. We would further suggest that inasmuch as this will be attended with the expenditure of money, this society make some provisions for requisite expenses. Respectfully submitted,

J. TAFT, J. E. ROBINSON, OTTO ARNOLD.

On motion of Dr. Sedgwick, a committee as suggested in the report, was then appointed as follows: Drs. J. Taft, J. R. Callahan, Otto Arnold.

Moved by Dr. Robinson that the members of this society be assessed $4 per member in order to defray the expenses of the above committee. After discussion the motion was withdrawn.

On motion of Dr. Arnold, a committee of two was appointed to solicit subscriptions to defray expenses of said committee. Drs. Arnold and Runyan were appointed as said committee.

On motion of Dr. J. Taft, 4 P. M. this day was set apart for the exhibition of appliances, and 8:30 to 10 A. M. to-morrow for clinics.

A paper was then read by Dr. Martha J. Robinson. Subject, Woman's Work in the Profession. Paper was discussed at length and with much enthusiasm by Drs. Butler, Smith, Wright, Taft and others.

Dr. Kempton then read a paper. Subject, One Way of Increasing a Dentist's Usefulness. Paper discussed by Drs. Callahan, Waye and others.

Dr. E. G. Betty then reported the proceedings of the 7th District Dental Society. Discussion of this report was postponed till the Committee on District Societies should report.

The following resolution was offered by C. I. Keely:

*Resolved*, That a committee of two be appointed to devise such means as will reach every dentist in the State soliciting subscriptions to defray expenses of Dental Law Committee. Said committee to have power to act. Resolution adopted.

The following committee was appointed: Dr. Otto Arnold, Dr. C. I. Keely. Adjourned.

AFTERNOON SESSION.

October 30, 1889. On motion, the reading of the minutes was dispensed with.

Committee on Dental Districts reported as follows:

The committee appointed at the Ohio State Dental Society at

Cincinnati last October, to investigate the advisability of organizing district societies throughout the State, and take such steps as they deemed avisable toward organization, make the following report: After looking well over the ground and fully considering the subject, your committee decided, that it was not only feasible, but eminently desirable and for the interest of the profession that such organizations be effected. It was further decided to divide the State into eight districts. Circular letters were sent to different dentists throughout the different districts urging a meeting and if possble the organization of a society in the respective districts. A constitution and by-laws was also arranged and suggested for adoption by the various district societies, and copies mailed with the circular letters. Reports were received from some of the districts stating that such an organization was not at present thought advisable in these respective districts. From others no replies were received and but two districts have so far decided upon organization. The 7th District or Southwestern Ohio Dental Society, organized at Dayton in May, 1889, and the 2d District or Northwestern Ohio Dental Society, a preliminary meeting having been held Monday evening, October 22, at Toledo, when committees were appointed and November 29, 1889, set as a day for permanent organization. Signed by L. P. BETHEL.

On motion of Dr. J. E. Robinson, the above report was approved and committee continued.

After some remarks regarding the requirements for membership in the district societies, Dr. Betty offered the following resolution:

*Resolved*, That the State Committee be advised to fix a uniform standard of admission to the district societies; this standard to be similar to that of membership in the State society. On motion, the above resolution was laid on the table.

Prof. H. A. Smith then spoke on the subject of Legal Liabilities of Corporations for injuries the result of negligence. The subject was passed after having been discussed by Drs. Kempton, Harroun and others.

Dr. W. H. Whistlar then read a voluntary paper on the

Chemical Composition of the Dental Pulp. After discussion and questions by Prof Smith the subject was passed.

The hour having arrived for the exhibition of appliances, Prof. J. Taft exhibited and explained the Evans Gold Crown and a number of other appliances. Also, a ready method of forming Gold Crowns by Dr. C. H. Rosenthal, of Cincinnati. Drs. Custer and Harrison and Robinson also exhibited appliances.

On motion, a vote of thanks was tendered Dr. Geo. Evans, of New York City, also Dr. C. H. Rosenthall, of Cincinnati, for their exhibits. Adjourned.

MORNING SESSION.

October 31, 1889. Society called to order at 10 A. M. by the President. Minutes of morning and evening sessions of 29th inst. read and approved.

Dr. H. H. Newton, of Cleveland, having been recommended by the Board of Directors for active membership was duly elected.

Dr. C. H. James, of Cincinnati, was duly elected to Honorary Membership.

On motion of Dr. Robinson, the motion to place Dr. H. H. Harrison, of Wheeling, on the list of Honorary Membership, was taken from the table. Dr. H. H. Harrison was then duly elected to Honorary Membership in this society.

The committee on necrology then presented the following reports:

IN MEMORIUM.

Your committee appointed to prepare and present resolutions expressive of the sense of loss experienced by the Association in the removal of some of its members by death, during the year just passed, present the following:

*Whereas*, It has been the will of our Heavenly Father, the allwise dispenser of events, to remove from this life our neighbor, friend, associate and professional brother, Dr. F. H. Rehwinkle of Chillicothe, O., it becomes us to bow with humble submission, though in sorrow, to this dispensation. He was a gentleman of the highest order, a man not only respected, but beloved by all who knew him. He was tender and gentle as a child, yet bold, strong and vigorous in the maintainance of the right. He possessed a moral nature of a high order. He occupied an enviable position as a public spirited citizen, always having in his charge, more or less, the responsibility of public affairs, and this not only of his own city and vicinity, but of the State and Country as well. In all his relations of life, public, professional, social and domestic, he was one to be emulated by all. In the possession of this combination of qualities, there is perhaps no where to be found his superior and but seldom his equal. Therefore,

*Resolved*, That we can not do less than, to thus give expression and put upon record, the evidence of our sorrow and sense of loss, in the removal of him who was one of the oldest, most active and efficient members of this society.

*Resolved*, That we most heartily commend his qualities, aspirations and activities of life as well worthy of emulation by all. We recommend that this preamble and resolutions be engrossed on a memorial page of the book of proceedings of this society. Also, that a copy properly prepared, be sent to the family of our departed brother.

Signed, J. TAFT. / C. R. BUTLER. / H. A. SMITH. } Committee.

Dr. A. Berry, of Cincinnati, died in Boston, Mass., October 12, 1889.

Dr. Berry was one of the signers, in 1866, of the circular issued to the profession of the State asking them to meet in Columbus for the purpose of organizing a dental society. He was continuously a member until his death. In 1883 he was chosen president. Always an earnest supporter of dental associations and especially of our State society, as well as a frequent contributor to dental literature. In the death of Dr. Berry our society and the profession at large have lost an able, honorable and enthusiastic associate. The Ohio State Dental Society, in annual session, desires to express its sincere sympathy with the friends and family of our deceased brother. The secretary is requested to make record of these proceedings, and send a copy to Mrs. Berry.

Signed, J. TAFT. / C. R. BUTLER. / H. A. SMITH. } Committee.

Dr. C. R. Taft, of Cincinnati, died June 15th, 1889.

Everybody who knew Dr. Taft, the quiet, unobtrusive gentleman, excellent dentist, the cheerful friend, loved him. Every one of us who knew him, knew that he was a man of the most sterling integrity. He was a man whom everybody trusted. He was president of this body in the year 1876 and was always a valuable member of this and other societies, as long as he was connected with them, and yet his distinguishing characteristic was a dignified modesty in the kindly yet strict performance of every duty of life. The lives of such men as Dr. C. R. Taft are successes in the highest sense of the meaning of the word success. Such lives make examples worthy of study and imitation by us all. We desire on this occasion to express our sympathy with his family in their bereavment.

Signed, H. A. SMITH. } Committee.
C. R. BUTLER. }

Dr. R. L. Evans, of Toledo, a member well and favorably known for many years, has also joined the ranks of the majority, where, with Keely, Rehwinkle, C. R. Taft and Berry, who have gone before, he will always hold a place in the hearts of the members of this society. He was a good dentist, a pleasant companion and a useful citizen; though he was not active in the work of the society as an officer, he always did his share of the work as a quiet member in the discussions and upon committees.

J. TAFT. }
Signed, H. A. SMITH. } Committee.
C. R. BUTLER. }

On motion, the above report was adopted and ordered spread upon the minutes.

After spending an hour in eulogistic remarks upon the great loss this society had sustained in the death of Drs. Rehwinkle, Taft, Berry and Evans, the society proceeded to the election of officers, with the following result:

President, W. H. Sedgwick, Granville; 1st Vice-President, Martha J. Robinson, Cleveland; 2nd Vice-President, E. G. Betty, Cincinnati; Secretary, J. R. Callahan, Cincinnati; Treasurer, C. I. Keely, Hamilton.

Drs. F. S. Maxwell and C. R. Butler were elected to fill the unexpired terms of Drs. Rehwinkle and Harrison on the Board of Directors. Directors for three years, Drs. E. G. Betty, Otto Arnold, Chas. Welch, F. C. Runyan.

Board of Examiners, L. E. Custer and J. Taft.

Drs. Harroun and Jennings were appointed a committee to conduct the newly elected president to the chair.

Dr. Sedgwick, after thanking the society for the honor conferred upon him, assumed the reins of government and proceeded with the business before the society.

On motion, it was decided to hold the next annual meeting of this society in the city of Columbus.

On motion, a vote of thanks was tendered Dr. J. Robinson for his exhibit of dental appliances. Also to the President and Committee of Arrangements; to the press of Cleveland; to the proprietors of Hollenden House.

The President stated that all voluntary papers would be received as property of the society.

On motion, the society adjourned to meet in the city of Columbus, Ohio, on the last Tuesday of October, 1890.

J. R. Callahan, 79 W. 8th St., Cincinnati, Secretary.

To Handle Small Thin Sections.—A very convenient trowel for this purpose is made by inserting the head of a large needle into a penholder, or other suitable handle, then filing the needle flat on two opposite sides and breaking off the point. Such an instrument does not take up as much fluid, with a small specimen, as the ordinary trowel does. It also has other advantages over the customary methods of handling small thin sections of either animal or vegetable tissne.

It is said that children born deaf and dumb can now be taught to speak. Mr. Pinel has constructed an electric screen, by which the sound is propagated by the action of the voice on the walls of the upper palate and larynx and communicated to the brain, which by dint of education may be comprehended.—*Sanitary Volunteer.*

